# mTOR and Tumor Cachexia

**DOI:** 10.3390/ijms19082225

**Published:** 2018-07-30

**Authors:** Adrian P. Duval, Cheryl Jeanneret, Tania Santoro, Olivier Dormond

**Affiliations:** Department of Visceral Surgery, Lausanne University Hospital, 1011 Lausanne, Switzerland; adrian.duval@chuv.ch (A.P.D.); cheryl.jeanneret@unil.ch (C.J.); tania.santoro@chuv.ch (T.S.)

**Keywords:** tumour cachexia, mTOR, signalling, metabolism, proteolysis, lipolysis

## Abstract

Cancer cachexia affects most patients with advanced forms of cancers. It is mainly characterized by weight loss, due to muscle and adipose mass depletion. As cachexia is associated with increased morbidity and mortality in cancer patients, identifying the underlying mechanisms leading to cachexia is essential in order to design novel therapeutic strategies. The mechanistic target of rapamycin (mTOR) is a major intracellular signalling intermediary that participates in cell growth by upregulating anabolic processes such as protein and lipid synthesis. Accordingly, emerging evidence suggests that mTOR and mTOR inhibitors influence cancer cachexia. Here, we review the role of mTOR in cellular processes involved in cancer cachexia and highlight the studies supporting the contribution of mTOR in cancer cachexia.

## 1. Introduction

Cancer is a leading cause of death worldwide with nearly 600,000 cancer related deaths projected to occur in 2018 in the United States [1]. Most cancer related deaths are observed in patients with metastasized tumours. Indeed, at least two factors contribute to lethality from metastasis. Firstly, tumour-secreted factors profoundly modify cancer patients’ homeostasis, increasing their susceptibility to infections and thrombo-embolic events, with major morbid and mortal consequences [2,3]. Secondly, organ invasion by neoplastic cells can lead to organ failure.

Tumour secreted factors promote tumour cachexia, a multifactorial condition characterized by decreased body weight due to losses of skeletal muscle and adipose tissue mass [4]. Nearly 80% of all cancer patients are affected by tumour cachexia, which significantly contributes to cancer-related morbidity and mortality. In particular, cachexia is associated with poor performance status, reduced tolerance to therapies and a high mortality rate [5,6]. The incidence of cachexia varies according to tumour type and is highly associated with cancers of the oesophagus, stomach, liver and lung. Cachexia may precede clinical diagnosis of cancer and may be present with small primary tumours. Since treatment options against tumour cachexia are so far very limited, a greater understanding of the underlying mechanisms is necessary.

The mechanistic target of rapamycin (mTOR) is an ubiquitously expressed serine-threonine kinase that is part of two different protein complexes named mTORC1 and mTORC2 [7]. Both complexes participate in cell proliferation and survival and, accordingly, represent a target in cancer therapy. Indeed, drugs which inhibit mTOR, named rapalogs, have proven clinical benefits in cancer patients and were approved for the treatment of different advanced neoplasias. Of note, mTORC1 is a major signalling intermediary, which stimulates anabolic processes, including protein, lipid and nucleotide synthesis and represses catabolic pathways such as autophagy. Emerging evidence demonstrated the importance of mTORC1 in stimulating skeletal muscle growth and in facilitating adipogenesis and lipogenesis [8]. This suggests that mTOR might be involved in tumour-related cachexia and, conversely, that mTOR inhibitors during cancer treatments might contribute to tumour cachexia.

Here, we review the putative roles played by mTOR in cellular processes relevant to tumour cachexia and highlight the experimental evidence of mTOR signalling importance in these processes. We further speculate on the consequences of mTOR inhibition in the development of cancer cachexia.

## 2. Cancer Cachexia

Patients with advanced forms of cancer are frequently affected by a multifactorial syndrome named cachexia. It results from a negative balance of energy caused by reduced caloric intake and altered metabolism including inflammation, elevated catabolism and excess energy expenditure [9,10]. Consequently, patients affected by cachexia present body weight loss, with predominant decrease of skeletal muscle and adipose tissue mass, eliciting reduced response to treatment, reduced quality of life and decreased survival. Altered energy balance is a major feature of tumour cachexia with reduced energy intake and increased resting energy expenditure [11]. Whereas the central nervous system is mainly responsible for reduced caloric intake, the increased energy expenditure relies on different causes, including tumour metabolism, inflammation and metabolic cycling [9].

**Figure 1:**
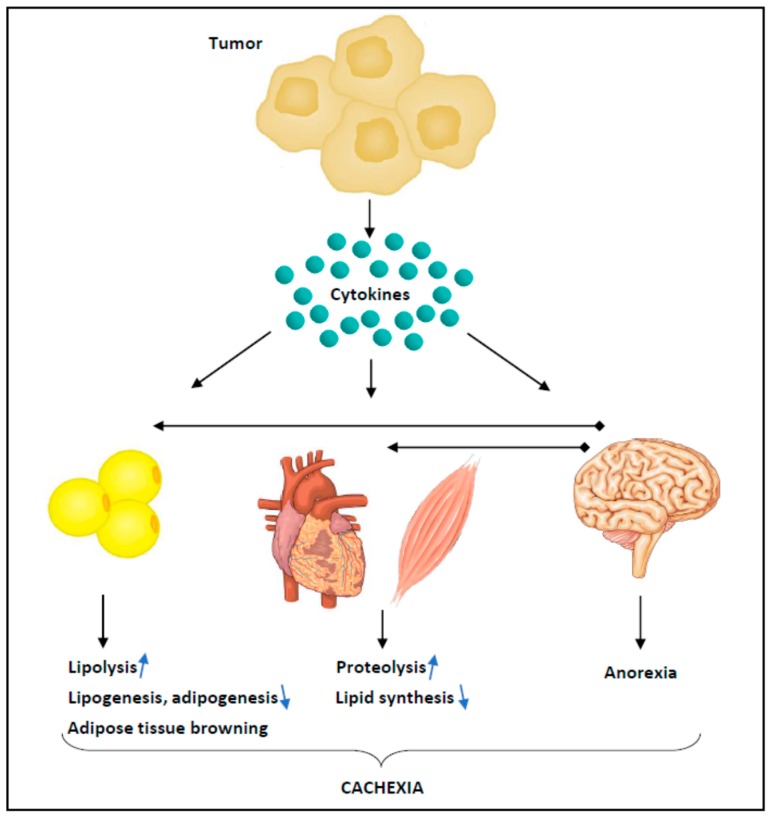
Mechanisms involved in cancer cachexia. Tumour-derived catabolic factors such as pro-inflammatory cytokines act on target tissues to elicit excess catabolism. Alteration of the central nervous system results in reduced food intake and increased catabolic neural outputs. Proteolysis is induced in skeletal and cardiac muscles through up-regulation of the ubiquitin-proteasome system and autophagy. Reduced protein synthesis has also been reported. Loss of adipose tissue results from increased lipolysis, decreased lipogenesis and adipogenesis and white adipose tissue browning.

Cachexia is driven by multiple mediators produced by cancer cells and cells within the tumour microenvironment [12]. Among these mediators are pro-inflammatory cytokines such as prostaglandin E_2_, IL-6, TNF, IFNγ, TRAF6, IL-1α, IL-1β and other tumour-derived catabolic factors such as activin and myostatin [12]. These molecules directly promote catabolism in target tissues including skeletal and cardiac muscles as well as adipose tissue (Figure 1). In addition, they produce central nervous system alterations leading to reduced caloric intake and increased catabolic neural outputs [9].

In skeletal muscle, the majority of these factors activate intracellular signals that lead to transcription of genes encoding components of the autophagy and ubiquitin-proteasome systems (UPS) [13]. Once activated, these systems selectively destroy myofibrillar proteins resulting in muscle atrophy [9]. Several studies highlighted the importance of UPS in degradation of muscular proteins [14,15,16,17]. Furthermore, more recent studies demonstrated increased proteasome activity in numerous murine models of cancer cachexia and showed that proteasome inhibitors improve cachexia in tumour bearing mice [18]. In contrast to pre-clinical studies, the role of UPS during loss of skeletal muscle in cancer patients is not clear. Whereas some studies showed increased expression of UPS components [19,20,21], others failed to detect any changes [22,23]. Besides UPS, autophagic processes contribute to protein degradation in tumour cachexia [24]. In C26 tumour bearing mice, autophagy is induced in muscular cells at initial and advanced stages of cachexia. Similar observations were made in mice transplanted with Lewis lung carcinoma and in rats bearing hepatomas [24], showing that this phenomenon was not specific of C26 tumours.

In addition to catabolic processes, reduced protein synthesis also participates in muscle atrophy in cancer cachexia [25,26,27]. Under physiological conditions, insulin-like growth factor 1 (IGF1)/PI3K/AKT/mTOR signalling pathway embodies the main anabolic pathway [28]. In the context of tumour cachexia however, contrasting results were reported as both increased and decreased AKT activity was observed in different models [29,30,31]. Hence, additional studies are needed to assess the role of reduced protein synthesis in cancer cachexia.

Besides loss of skeletal muscle, cardiac muscle atrophy is also associated with cancer cachexia [32]. The pathogenesis of cardiac atrophy remains poorly explored but seems to share similar mechanisms with skeletal muscle atrophy. Indeed, reduced protein synthesis and increased protein degradation in hearts of cachectic rodents were detected [33,34]. In addition, cardiomyocytes apoptosis was also observed in AH-130 tumour-bearing rats and C26 tumour bearing mice cachectic models [35,36].

Finally, as mentioned previously, tumour cachexia is also characterized by loss of adipose tissue [37]. In contrast to skeletal muscle loss, little is known about the role of fat shrinkage in cancer. Nevertheless, an association between fat loss and poor outcomes was identified in advanced cancer patients [38,39]. Several mechanisms are responsible for the loss of adipose tissue including reduced food intake, increased lipolysis, decreased lipogenesis, impaired adipogenesis and decreased lipid deposition [37,40]. In particular, lipolysis represents a major cause of adipose tissue loss in cancer, as cachectic cancer patients present increased expression of hormone sensitive lipases compared to weight stable cancer patients [41,42]. In addition, cachectic patients exhibit increased expression of receptors of lipolytic hormones on adipocytes [42]. Furthermore, besides hormone-sensitive lipases, adipose triglyceride lipase (ATGL) contributes to lipolysis in cancer patients, as ATGL-deficient tumour bearing mice did not show increased lipolysis [43].

Enhanced lipolysis generates excess fatty acids that are subsequently oxidized by mitochondria. Accordingly, up-regulation of genes regulating mitochondrial lipid oxidation was observed in animal models and in patients with cachexia [40,44]. Additionally, recent studies also found that white adipose tissue browning contributes to fatty acid catabolism [45,46]. This process uncouples mitochondrial respiration toward thermogenesis instead of ATP synthesis, resulting in increased lipid mobilization and energy expenditure [47].

Finally, in addition to augmented lipolysis and fat oxidation, loss of fat mass in cancer patients relies on reduced lipid deposition and lipogenesis. Decreased activity of fatty acid synthase and lipoprotein lipase was shown in adipose tissue of cancer patients [48] and adipogenesis, a process essential to form mature adipocytes, is impaired in experimental models of cancer cachexia with reduced expression of adipogenic transcription factors [44,49,50].

## 3. mTOR Signalling Pathway

**Figure 2:**
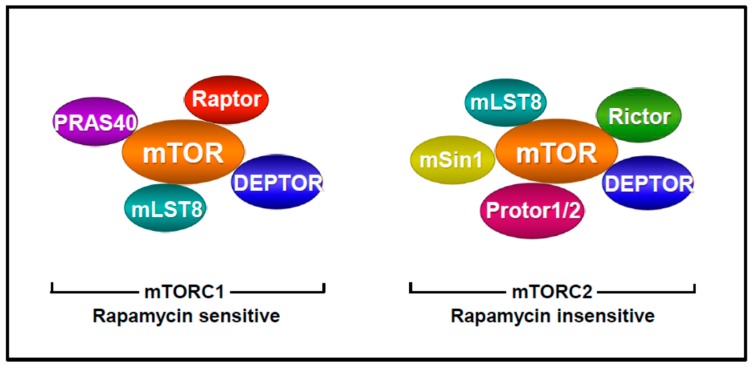
Components of mTORC1 and mTORC2. Specific components of mTORC1 are Raptor and PRAS40 and specific components of mTORC2 are Rictor, mSin1 and Protor1/2.

The mechanistic target of rapamycin (mTOR) is an ubiquitously expressed and well conserved serine/threonine kinase belonging to the PI3K-related kinases family [7]. mTOR is one of the main component of two protein complexes named mTOR complex 1 and mTOR complex 2 (respectively mTORC1 and mTORC2), which are involved in cell growth regulation (Figure 2) [7].

**Figure 3:**
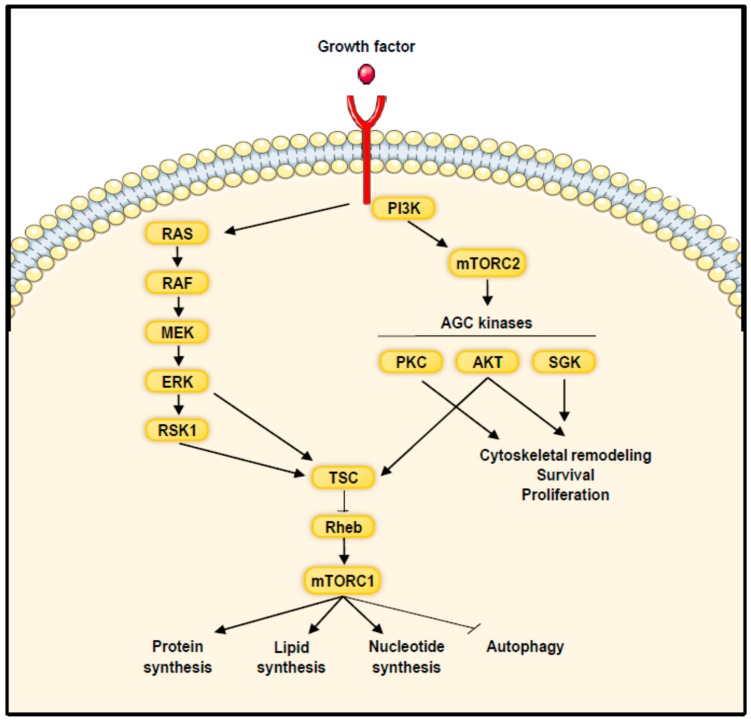
Activation of mTOR signalling pathway by growth factors. Upon stimulation of growth factor receptors, mTORC1 is activated via the PI3K/AKT and Ras/Raf/Mek/Erk signalling pathways and stimulates anabolic processes and represses autophagy. mTORC2 activation requires PI3K. Once activated mTORC2 regulates cytoskeletal organization, cell proliferation and survival by phosphorylating members of the AGC kinases family.

mTORC1 is composed of mTOR, raptor (Regulatory-associated protein of mTOR) [51], mLST8 (mammalian lethal with Sec13 protein 8) [52] and two inhibitory proteins PRAS40 (proline-rich AKT substrate 40 kDa) [53] and Deptor (Dishevelled, Egl-10 and Pleckstrin domain-containing mTOR-interacting protein) [54]. In presence of favourable extracellular conditions, mTORC1 coordinates cell growth by stimulating protein, lipid and nucleotide synthesis and repressing autophagy [7]. Several factors regulate mTORC1 activity including growth factors, hormones, amino acids, energy level, oxygen and stress. The intracellular signalling pathways leading to mTORC1 activation by growth factors and hormones were identified and can be summarized as follows (Figure 3). Ensuing binding and activation of their specific receptors, two major signalling pathways are stimulated; the Ras/Raf/Mek/Erk as well as the PI3K/AKT signalling pathways. In turn, activated AKT or Erk and its downstream effector p90^RSK^ phosphorylate tuberous sclerosis complex 2 (TSC2) resulting in its dissociation from TSC1 and TBC1D7. This results in the inactivation of the TSC complex by dissociation from the lysosomal membrane where it exerts its inhibition on the GTPase Rheb [55,56,57,58,59]. Since the TSC complex converts the GTPase Rheb to its inactive form, TSC complex inhibition elicits Rheb activation, which strongly enhances mTORC1 activity. In addition to its effect on TSC2, AKT enhances mTORC1 activity by phosphorylating and inactivating PRAS40 [53]. Besides growth factors, energetic modulations also regulate mTORC1 activity via the TSC axis [60]. Indeed, reduced levels of ATP following energy deprivation lead to the activation of the AMP-activated protein kinase (AMPK), which in turn activates TSC2 by phosphorylation, resulting in enhanced inhibition of Rheb and consequently of mTORC1. Moreover, AMPK reduces mTORC1 activity by phosphorylating raptor [61]. Hypoxia also downregulates mTORC1 activity either by activating AMPK or by inducing the expression of REDD1 which inactivates mTORC1 by activating the TSC complex [62]. Finally, mTORC1 activity is regulated by amino acid levels. In this context, amino acids signalling to mTORC1 involve recruitment of mTORC1 at the surface of the lysosomes. In turn, mTORC1 associates with Rag GTPases which promote its interaction with the lysosomal pool of Rheb [63,64].

Once activated mTORC1 regulates diverse cellular functions needed for cell growth and proliferation. In particular, mTORC1 promotes protein synthesis directly by phosphorylating S6K1 and 4EBP. S6K1 phosphorylates several substrates to promote mRNA translation initiation [65]. Phosphorylation of 4EBP by mTORC1 leads to its dissociation from elF4E and results in 5′ cap-dependent mRNA translation [66]. Besides protein, mTORC1 also promotes nucleotide synthesis required for DNA replication and up-regulates glycolysis, leading to newly generated biomass [67,68,69]. Finally, and as discussed in more detail later, mTORC1 stimulates lipid synthesis [70].

Interestingly, activated mTORC1 is able to signal back to the plasma membrane to inhibit growth factor signalling, protecting from pathway over activation [71]. Two different mechanisms were identified in this process. Firstly, mTORC1 and S6K1 promote insulin receptor substrate-1 degradation [72]. Secondly, mTORC1 stabilizes Grb10, which acts as an endogenous inhibitor of receptor tyrosine kinases [73,74].

mTORC2 comprises mTOR, Rictor (rapamycin insensitive companion of mTOR) [75], mLST8, DEPTOR, mSin1 (mammalian stress-activated protein kinase-interacting protein) [76,77] and Protor1/2 (protein observed with rictor 1 and 2) (Figure 2) [78]. Upstream regulators of mTORC2 are mainly growth factors and PI3K [79]. Upon binding of the PH domain of mSin1 to phosphoinositides generated by PI3K, the inhibitory effect of mSin1 PH domain on mTORC2 is relieved, leading to mTORC2 activation [80]. Additionally, PI3K stimulates mTORC2 activity by inducing its association to ribosomes, which probably represent another cellular pool of mTORC2 [81]. Following activation, mTORC2 functions as a kinase of several members of the AGC family of protein kinases, including PKC, AKT and SGK1 [79]. By activating members of the PKC family including PKCα, PKCδ, PKCγ and PKCζ, mTORC2 regulates cytoskeletal remodelling and cell migration [75,82,83,84]. More importantly, mTORC2 also phosphorylates AKT, which regulates cell proliferation and survival and appears to provide substrate specificity to AKT [85].

Several genetic mutations have been reported that lead to PI3K/AKT/mTOR pathway activation in cancer. Activating mutations of PI3K, AKT, mTOR as well as inactivating mutations of TSC1, TSC2 or PTEN are commonly observed in several types of cancers [86,87]. More recently, an extensive study of more than 11,000 human cancers confirmed the high prevalence of genetic mutations of components of mTOR signalling pathway [87]. In addition, activation of the pathway without genetic mutations was also found suggesting alternate mechanisms for pathway activation [87].

Since mTOR signalling controls both cell growth and proliferation and since activating mutations of components of this pathway are frequently found in cancer, many studies addressed the effects of mTOR inhibitors in cancer therapy [88,89,90]. Initially, mTOR inhibition was achieved with rapamycin or its derivatives named rapalogs. Rapamycin associates to FKBP12, which bind together to the FRB domain of mTOR, partially occluding the access of substrates to its kinase domain [91]. Inhibition of mTORC1 by rapalogs is however incomplete as some protein residues phosphorylated by mTORC1 are rapamycin resistant [92]. Furthermore, rapalogs do not provide an immediate inhibition of mTORC2. Indeed, mTORC2 is classically rapalog-insensitive in most cancer cell types [93]. Only a limited number of cell types with prolonged exposure to rapalogs show mTORC2 inhibition, presumably from the inability to generate novel mTORC2 complexes from rapalog-bound mTOR [94]. To overcome these limitations, a second generation of mTOR inhibitors was developed to directly target the kinase domain of mTOR. Accordingly, compared to rapalogs, kinase inhibitors of mTOR inhibit mTORC2 and provide a complete inhibition of mTORC1 [95].

To date only rapalogs are approved for the treatment of various advanced cancers including renal cell carcinoma [96,97], advanced pancreatic neuroendocrine tumours [98], postmenopausal hormone receptor-positive advanced breast cancer in combination with exemestane [99], advanced non-functional neuroendocrine tumours of the lung or gastrointestinal tract [100] and refractory mantle cell lymphoma [101]. The anti-cancer efficacy of rapalogs is however limited failing to provide long lasting benefits.

## 4. mTOR in Muscle and Lipid Metabolism

mTOR signalling is an important anabolic pathway in skeletal muscle growth [102]. Indeed, genetic and pharmacologic experiments support a major role of mTOR in this process. Muscle-specific deletion of mTOR causes weight loss with a strong decrease of fast-twitch glycolytic muscles leading to premature death [103]. mTOR deficient muscles further display metabolic alterations including decreased oxidative capacity, altered mitochondrial regulation and glycogen accumulation. A similar phenotype is observed in raptor but not in rictor deficient muscles, indicating that mTORC1 disruption likely accounts for these changes [104]. Consistent with these findings, muscle depletion of S6K1, a direct downstream target of mTORC1, causes muscle atrophy [105]. Interestingly, chronic activation of mTORC1 also results in muscle atrophy and low body mass [106]. In this case, loss of muscle mass is primarily due to inhibition of autophagy by mTORC1 activity. Besides genetic gain and loss of function experiments, the role of mTOR signalling in skeletal muscle was demonstrated with chemical inhibition of mTOR. For instance, skeletal muscle hypertrophy induced by muscle overload was inhibited by rapamycin [107]. Of note, rapamycin does not induce muscle atrophy in control muscles. Rapamycin also inhibits muscle growth induced by the expression of a constitutive active mutant of AKT [108]. Early studies identified IGF-I and leucine as a major stimulator of mTORC1 in skeletal muscle [28,109]. In addition, mechanical stimulus promotes mTORC1 activity in part independently of IGF-I [110,111]. In particular, mechanical stimulation induced multisite phosphorylation of raptor resulting in up-regulated mTORC1 activity, promoting the lysosomal association of mTOR and abolishing the lysosomal association of TSC2 [112].

mTOR also influences various aspects of lipid metabolism including lipogenesis, adipogenesis, lipolysis and lipid oxidation [70]. mTORC1 is a particularly important mediator of lipid biogenesis by controlling the expression of many lipogenic genes. Sterol regulatory element-binding proteins (SREBPs) are components of a family of transcription factors that induce lipid synthesis and that are positively regulated by mTORC1 [113,114]. Several studies demonstrated that rapamycin decreases the expression of lipogenic genes by affecting SREBPs processing and activation [115,116,117]. Depletion of raptor but not rictor downregulates the expression of lipogenic genes confirming that mTORC1 and not mTORC2 is mainly involved in this process [116]. In contrast to these observations, recent studies highlighted the critical role of mTORC2 in lipid synthesis. Liver depletion of rictor results in reduced SREBP activity and expression of lipogenic genes [118,119]. On top of lipogenesis, mTOR is also involved in adipogenesis regulation. Indeed, rapamycin inhibits adipocyte differentiation in vitro [120,121] and adipogenesis was also abrogated in 3T3-L1 preadipocytes following raptor deletion [122]. Likewise, the potential of raptor null mouse embryonic fibroblasts to differentiate into adipocytes is impaired [122]. The role of mTORC1 in adipogenesis was also addressed in vivo; specific knock-down of raptor in adipocytes limits lipid accumulation in adipocytes and protects mice from obesity induced by diet [122]. Similarly, rapamycin treated mice accumulate less adipose tissue [123,124]. However, although AKT plays an important role in adipogenesis, deletion of rictor in adipose tissue does not affect adipose tissue accumulation [125,126,127,128]. Hence, mTORC2-mediated AKT phosphorylation on Ser473 is not necessary for AKT to transmit pro-adipogenic signals. Nevertheless, deletion of rictor in white adipocyte progenitors is associated with less adipose tissue suggesting that mTORC2 is required for early adipogenesis [129].

mTOR further participates in adipose mass accumulation by inhibiting catabolic processes such as lipolysis. Indeed, circulating free fatty acids are elevated in humans treated with rapamycin [130] and higher lipolysis intensity was recorded in isolated adipocytes treated with rapamycin [131,132]. Finally, increasing mTORC1 activation via overexpression of Rheb inhibits lipolysis in 3T3-L1 adipocytes [131]. Regarding mTORC2, its activity also affects lipolysis, but contrasting results were found regarding the role of rictor in this process [127,128].

## 5. mTOR and Tumour Cachexia

mTORC1 involvement in tumour cachexia was evidenced in *Apc^Min^*^/*+*^ mice, a model of colorectal cancer that develops cachexia that is dependent on interleukin-6 [133]. Analysis of the gastrocnemius muscle of *Apc^Min^*^/*+*^ mice revealed a progressive decrease of mTORC1 activity from the initiation of cachexia to extreme body weight loss [134]. mTORC1 inhibition was mediated via the activation of AMPK by IL-6, which was further confirmed in C2C12 myoblasts [135]. This study suggests that reduction of anabolic mTORC1 signalling in skeletal muscle contributes to loss of muscle mass during cachexia. Accordingly, treadmill exercise restoring mTORC1 activity in skeletal muscle prevents cachexia in *Apc^Min^/^+^* mice [136]. The anti-cachectic role of mTORC1 was further substantiated in mice bearing Lewis cell carcinoma. In this model, cachexia was also associated with reduced mTORC1 signalling in the gastrocnemius muscle [137]. Furthermore, in vitro in C2C12 myoblasts, studies showed that stretch-induced mTORC1 activation was inhibited by media containing cachectic factors derived from Lewis cell carcinoma [138]. Finally, salidroside, a major phenylpropanoid glycosides found in *Rhodiola rosea* L., prevented tumour cachexia in CT-26 colon cancer and Lewis lung carcinoma and restored levels of muscle phospho-mTOR, used as a read-out of mTORC1 activity [139]. Hence, in these models, prevention of tumour cachexia is associated with restored mTORC1 activity. Finally, as mentioned earlier, activation of UPS is a major process that leads to skeletal muscle loss in cancer cachexia [10]. Recently, two studies demonstrated that mTOR inhibition leads to proteolysis via the UPS, suggesting that mTOR prevents protein loss by repressing anabolic processes [140,141]. Nevertheless, additional studies are needed to investigate it in the context of tumour cachexia.

In contrast to these results, mTORC1 inhibition was also reported to prevent loss of muscle mass in tumour cachexia [142]. In fact, colon cancer tumour-bearing mice and tumour patients display altered autophagic markers, suggesting that autophagy flux proceed at a slower rate. Pharmaceutical intervention with rapamycin in these mice restored autophagy in skeletal muscles and prevented tumour cachexia [142]. Similarly, treatment with an AMPK activator or aerobic exercise counteracted tumour cachexia-induced weight loss which was associated with increased autophagy. In addition, rapamycin prevented C2C12 myoblasts atrophy induced by colon carcinoma preconditioned media. This effect was abrogated following inhibition of autophagy, further suggesting that rapamycin-induced autophagy prevents loss of muscle during tumour cachexia [142]. Consistent with these observations, it was demonstrated that muscle-specific deletion of a crucial autophagy gene, Atg7, resulted in profound muscle atrophy and exacerbated muscle loss during denervation and fasting [143]. Taken together, these results suggest that autophagy can prevent muscle loss during tumour cachexia and that targeting mTORC1 to induce autophagy represent a treatment strategy to prevent cachexia.

Besides inducing autophagy, another mechanism was proposed to explain the anti-cachectic properties of mTOR inhibitors. In a transgenic murine lymphoma model, mice developed a cachectic syndrome characterized by reduced appetite, severe body weight loss, complete depletion of adipose tissue mass and significant loss of muscle mass [144]. This phenotype was associated with increased levels of cachexia mediators in particular interleukin-10. Administration of rapamycin in these mice prevented the development of cachexia and decreased IL-10 levels, suggesting that the production of pro-cachectic factors are regulated by mTOR. In particular, rapamycin improved appetite and reduced the severity of fat loss. Similarly, everolimus, a specific mTOR inhibitor, reduced IL-6 levels and alleviated the cachectic phenotype of CT-26 colon cancer bearing mice in which IL-6 is the main cachectic driver [144,145]. CT-26 tumours induced a significant decrease in the weight of tibialis anterior, gastrocnemius-soleus-plantaris complex and quadriceps muscles, which was prevented by everolimus treatment. Of note, everoliums did not induce muscle loss in non-tumour bearing control mice [145]. Therefore, suppression of cytokine production by targeting mTOR represents a treatment strategy to ameliorate tumour cachexia.

The effect of mTOR inhibition on tumour cachexia in cancer patients remains poorly investigated. Nevertheless, a retrospective study analysed the consequences of long-term treatment with rapalogs on cancer patients’ muscle mass. Twenty patients, treated with rapalogs as monotherapy for at least 6 months, were investigated by CT-scan [146]. A significant decrease of skeletal muscle area without affecting body weight nor adipose tissue was observed in these patients. However, as this study did not involve an untreated control group of patients, the presence of other regulating factors cannot be excluded. This study suggests at least that cancer patients treated with mTOR inhibitors do not experience adipose tissue loss.

Pivotal phase III studies that tested rapalogs in cancer patients did not address specifically cancer cachexia. Nevertheless, parameters that are associated with cancer cachexia were reported. In a multicentre double-blind study patients with advanced pancreatic neuroendocrine tumours were randomly assigned to the rapalog everolimus, 10 mg daily, or placebo. Two hundred and four patients received everolimus versus 203 placebos. Sixteen percent of patients receiving everolimus experienced weight loss compared to 4% in the placebo group [98]. Decreased appetite was also more frequently reported in the everolimus group (20% vs. 7%). Similar findings were found in a phase III randomized trial comparing everolimus with exemestane to placebo with exemestane in patients with hormone receptor positive advanced breast cancer [99]. Of the 485 patients in the everolimus group, 19% had decreased weight versus 5% in the placebo group. In addition, 29% displayed decreased appetite under everolimus treatment compared to 10% of patients receiving placebo. Reduced appetite in patients treated with rapalog was observed in three additional phase III studies [100,101,147]. Taken together, these results show that patients treated with mTOR inhibitors present more frequently signs and symptoms that are either part of tumour cachexia or specific to mTOR inhibitors and in this case, that may worsen cachexia. In addition, they further show that some patients are more sensitive to the side effects generated by mTOR inhibitors. Hence, in the context of cachexia, it will be important to be able to detect these patients early in the course of treatment.

## 6. Conclusions

Tumour cachexia, characterized by weight loss due to decreased skeletal muscle and lipid mass, is a severe condition in cancer patients with limited therapeutic options. Initial studies demonstrate the complex and contrasting role played by mTOR in this process. On one hand, mTORC1 activity is significantly reduced in skeletal muscles and lipid tissue of cachectic mice suggesting that loss of mTORC1 activity results in reduced protein and lipid synthesis. On the other hand, inhibition of mTORC1 protects from tumour cachexia by up-regulating autophagy and by inhibiting production of pro-cachectic factors. Hence mTORC1 plays a dual role in tumour cachexia that needs to be fully characterized. In addition, clinical trials that specifically address the effects of mTOR inhibitors on tumour cachexia are needed.

## Abbreviations

4EBP	Eukaryotic translation initiation factor 4E-binding protein
AMPK	AMP activated protein kinase
Erk	Extracellular signal-regulated kinase
Grb10	Growth factor receptor-bound protein 10
IL	Interleukin
IGF-1	Insulin-like growth factor-1
mTOR	Mechanistic target of rapamycin
PI3K	Phosphatidylinositol 3-kinase
Raptor	Regulatory associated protein of mTOR
Rheb	RAS homolog enriched in brain
Rictor	Rapamycin insensitive companion of mTOR
TSC	Tuberous sclerosis complex
UPS	Ubiquitin proteasome system
